# Prediction of CD3 T cells and CD8 T cells expression levels in non-small cell lung cancer based on radiomic features of CT images

**DOI:** 10.3389/fonc.2023.1104316

**Published:** 2023-02-13

**Authors:** Lujiao Chen, Lulin Chen, Hongxia Ni, Liyijing Shen, Jianguo Wei, Yang Xia, Jianfeng Yang, Minxia Yang, Zhenhua Zhao

**Affiliations:** ^1^ Department of Radiology, Shaoxing People’s Hospital, Shaoxing, Zhejiang, China; ^2^ Department of Ultrasound, Affiliated hospital of Shaoxing University, Shaoxing, Zhejiang, China; ^3^ Department of Pathology, Shaoxing People’s Hospital, Shaoxing, Zhejiang, China; ^4^ Department of Radiology, Shaoxing Maternal and Child Health Hospital, Shaoxing, Zhejiang, China

**Keywords:** non-small-cell lung cancer, radiomics, model, CD3, CD8

## Abstract

**Background:**

In this work, radiomics characteristics based on CT scans were used to build a model for preoperative evaluation of CD3 and CD8 T cells expression levels in patients with non-small cell lung cancer (NSCLC).

**Methods:**

Two radiomics models for evaluating tumor-infiltrating CD3 and CD8 T cells were created and validated using computed tomography (CT) images and pathology information from NSCLC patients. From January 2020 to December 2021, 105 NSCLC patients with surgical and histological confirmation underwent this retrospective analysis. Immunohistochemistry (IHC) was used to determine CD3 and CD8 T cells expression, and all patients were classified into groups with high and low CD3 T cells expression and high and low CD8 T cells expression. The CT area of interest had 1316 radiomic characteristics that were retrieved. The minimal absolute shrinkage and selection operator (Lasso) technique was used to choose components from the IHC data, and two radiomics models based on CD3 and CD8 T cells abundance were created. Receiver operating characteristic (ROC), calibration curve, and decision curve analyses were used to examine the models’ ability to discriminate and their clinical relevance (DCA).

**Results:**

A CD3 T cells radiomics model with 10 radiological characteristics and a CD8 T cells radiomics model with 6 radiological features that we created both demonstrated strong discrimination in the training and validation cohorts. The CD3 radiomics model has an area under the curve (AUC) of 0.943 (95% CI 0.886-1), sensitivities, specificities, and accuracy of 96%, 89%, and 93%, respectively, in the validation cohort. The AUC of the CD8 radiomics model was 0.837 (95% CI 0.745-0.930) in the validation cohort, with sensitivity, specificity, and accuracy values of 70%, 93%, and 80%, respectively. Patients with high levels of CD3 and CD8 expression had better radiographic results than patients with low levels of expression in both cohorts (p<0.05). Both radiomic models were therapeutically useful, as demonstrated by DCA.

**Conclusions:**

When making judgments on therapeutic immunotherapy, CT-based radiomic models can be utilized as a non-invasive way to evaluate the expression of tumor-infiltrating CD3 and CD8 T cells in NSCLC patients.

## Introduction

The most frequent cancerous condition, lung cancer is one of the main causes of cancer-related death globally. Nearly 80% to 85% of lung cancers are non-small cell lung cancer (NSCLC), and more than 50% of patients have distant metastases when they are diagnosed. Despite advancements in early diagnosis and novel treatment methods, five-year survival rates remain at 10–20% (1). Surgical resection continues to be the gold standard of treatment for lung cancer. The identification of immune checkpoint molecules, such as programmed death-1 (PD-1) and programmed death-ligand 1 (PD-L1) and cytotoxic T lymphocyte-associated antigen-4 (CTLA-4), has made immunotherapy one of the most promising curative modalities for lung cancer in recent years. Immunotherapy combined with surgery has considerably increased the survival rate of patients with lung cancer. Immune checkpoint inhibitor therapy has a 17–21% cure rate for patients with stage IV non-small cell lung cancer (2). The effectiveness of immunotherapy is primarily influenced by the tumor immune microenvironment (TIME) phenotype, particularly CD8 T cells infiltration into tumors, which positively correlates with immunotherapy efficacy and survival (3–6). The main immune cells that carry out immune surveillance are CD8 T cells. When T-cell receptors (TCR) are recognized, CD8 T cells get activated, multiply quickly, and develop into cytotoxic T lymphocytes (CTL), which kill cancer cells when they come into contact with them (7). The role of CD8 tumor-infiltrating lymphocytes (TILs) in the response to anti-PD-1/PD-L1 therapy has been demonstrated. A crucial prognostic factor and predictor for non-small cell lung cancer is CD8 TILs (8). High CD3 T cells counts have been found to be an independent predictor of response to PD-1 blockade, and checkpoint inhibitor immunotherapy against the programmed death protein-1 (PD-1)/programmed death-ligand 1 (PD-L1) immune axis has become the standard of care for advanced non-small cell lung cancer (9). Absolute CD3 T cells counts rather than percentages of each lymphocyte subpopulation are a better predictor of a patient’s immunological state. Longer progression-free survival is associated with higher overall CD3 T cells counts (10). Nearly all T lymphocytes express CD3 T cells, which are biomarkers of T lymphocytes and have anti-tumor activity, prognostic importance for overall survival and recurrence rate, and are important prognostic predictors (11–13). The identification of CD3 and CD8 T cells in tumor lesions may aid in predicting the course of the tumor and the prognosis of the patient.

The assessment of tumor CD3 and CD8 T cells infiltration currently relies on tissue specimens, but acquiring tissue samples necessitates intrusive procedures such surgical procedures or needle biopsies, making it unable to perform dynamic and repetitive observations. Additionally, local specimens frequently do not represent the entire tumor due to the diverse development of malignancies. A non-invasive and repeatable method to evaluate the infiltration of tumor CD3 and CD8 T cells is clinically desirable due to the drawbacks of the aforementioned techniques. Radiomics is a discipline with a lot of room for growth. It has advanced quickly in recent years and produced superior outcomes in terms of illness diagnosis and differential diagnosis, tumor staging and grading, genotype prediction, choice of treatment strategy, efficacy assessment, and prognosis prediction (14).It has demonstrated notable benefits in treating lung cancers in particular (15).

Therefore, the purpose of this work is to determine whether it is possible to develop imaging histology labels using traditional CT image texture features in order to forecast the levels of CD3 and CD8 expression in NSCLC tumors. Our findings might make it easier to pinpoint patients who would respond well to immunotherapy.

## Materials and methods

### Patients

A total of 200 patients with pathologically diagnosed NSCLC who received chest CTs at our institution between 2020 and 2021 were gathered. Finally, 105 lung cancer patients who met the following criteria were included in the study: inclusion standards: (1) pathologically verified NSCLC; (2) a chest CT scan within one month of the procedure or biopsy; (3) the absence of any prior anticancer therapy; (4) the absence of any prior history of other malignant tumors; (5) full clinical information. Exclusion criteria include (1) patients with co-morbidities of other primary malignancies. (2) patients with poor imaging scan quality or unclear tumor boundaries; (3) patients with pure ground glass nodules; and (4) patients with no clinical information. Clinical record retrieval was used to acquire clinicopathological data, such as age, gender, pathological stage, location, key subtypes of pathology, and tumor size. The Shaoxing People’s Hospital study was authorized by the ethics committee.

### CT examinations

The chest was scanned using a Philips Brilliance 64-layer spiral CT scanner. The layer thickness was 1.25 mm, the tube current was 160 mAs, and the tube voltage was 120 kV. Iohexol, a contrast agent containing 300 mg/ml of iodine, was injected into the elbow vein during an enhancement scan at a dosage of 1.0-1.5 mL/kg at a rate of 3 mL/s with a latency of 25–30 s. After inspiration, the scan was held with the patient lying on his or her back with both hands on each side of the head. After inspiration from above the lung tip to below the diaphragm level, the patient was positioned in a supine posture with both hands on each side of the head. Images from the CT scan were exported in DICOM format.

### Immunohistochemical evaluation

**Figure 1:**
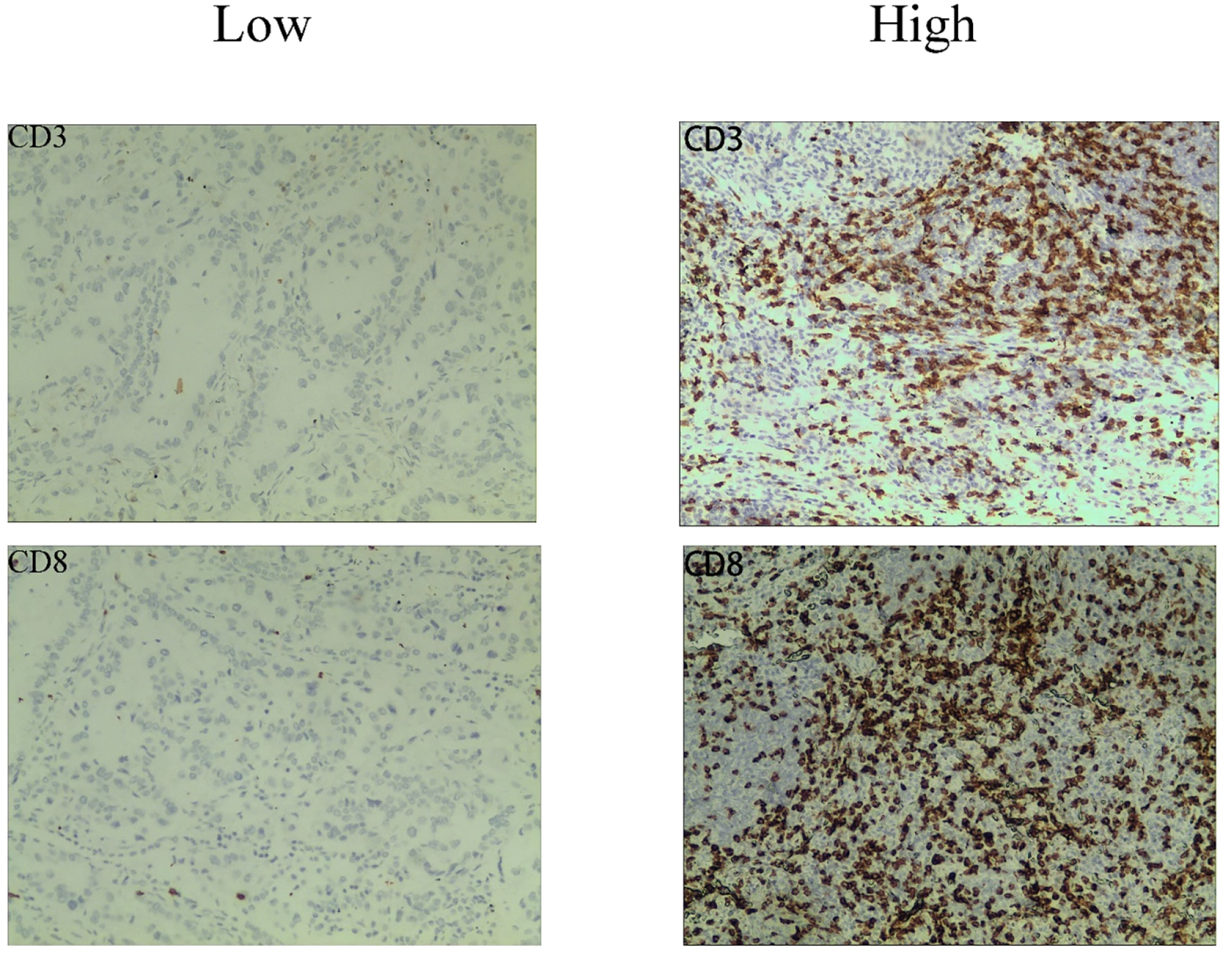
Representative immunohistochemistry staining images of CD3 and CD8 cells from NSCLC patients.

Immunohistochemistry (IHC) was used to analyze CD3+, and CD8+ T cells in paraffin-embedded tissue samples acquired by surgery or biopsy. The IHC protocol was followed throughout this surgery. Serial histological tumor sections of 4 μm thickness were obtained from formalin-fixed and paraffin-embedded tumor tissues and stained with rabbit anti-CD3 monoclonal antibody (1:200, GT219107, Shanghai, China) and, mouse anti-CD8 monoclonal antibody (1:200, GT211207, Shanghai, China) for IHC. Antibody labeling was detected with diaminobenzidine (DAB) and counterstained with hematoxylin. After dehydration, transparency, and sealing, the slides were observed by microscopy. The immunoreactivity score (IRS) was used to evaluate the staining results (15). The details were as follows: staining intensity (SI) level: 0, no staining; 1, light yellow; 2, brownish yellow; 3, dark brown; percentage of stained cells (PP) between 0 and 3 according to **Table 1** (16, 17).IRS = SI × PP, IRS < 2 was the low expression group and 2-9 was the high expression group (**Figure 1**). The results will be interpreted by a pathologist with 10 years of experience.

**Table 1:** Criteria of the level of positive cells.

Label	Percentage of stained cells			
	0	1	2	3
CD3	< 25%	25% -50%	51% -75%	>75%
CD8	< 10%	10% -30%	31% -60%	>60%

### Texture feature analysis Image segmentation

Radiomics used GE’s specialized software workstation AK to evaluate the data (Artificial Intelligence Suite, GE Healthcare, China). The lung window series of CT cross-sectional images was used to place all ROIs. The initial doctor traced a layer-by-layer outline of the whole tumor. The second doctor looked at the outline drawing made by the first doctor and used ITK-snap (version 3.8.0) to create a 3D ROI by defining each layer of the tumor. Enhanced CT mediastinal window images were used as a guide throughout the outlining procedure to help distinguish between the mass and nearby structures. The following criteria must be met while contouring a tumor: (1) trace the tumor margin; (2) include regions of ulceration, necrosis, and bleeding; and (3) avoid neighboring organs.

### Feature extraction and selection

The segmented field of view was used to extract 1316 texture-based radiomics features using AK, including 32 first-order features, 18 histogram features, 14 shape features, 24 gray level co-occurrence matrix (GLCM) features, 16 gray level run-length matrix (GLRLM), 16 gray level size zone matrix (GLSZM), 14 gray level dependence matrix (GLDM), and 5 neighborhood gray tone difference matrix (NGTDM) features. 186 features were extracted using the Laplace-Gaussian (LoG) transformation, 279 features were extracted using the Local Binary Pattern (LBP) filtering, and 744 features were extracted using the wavelet transformation based on eight filter channels. The same number of first-order grayscale statistical features and texture features were also extracted using variously transformed images. All patients were randomly assigned to one of two cohorts after feature extraction: a training cohort and a validation cohort (7:3). We next performed a number of data preprocessing and feature selection methods to standardize the data and eliminate redundant information. Following Z-Score normalization and redundant feature removal with ANOVA-KW and correlation analysis, missing values were initially interpolated using the median of the full feature column. Finally, data dimensionality reduction on the imaging histology features is carried out using the least absolute shrinkage and selection operator (LASSO) and 10-fold cross-validation. The final CD3 and CD8 models only left 10 and 6 optimal features, respectively, with the final models leaving 10 features (2 GLDM, 1 GLRLM, 3 GLSZM, 3 GLCM, 1 SHAP) and 6 features (2 GLDM, 1 GLRLM, 2 GLSZM, 1 first order).

### Model establishment and validation

**Figure 2:**
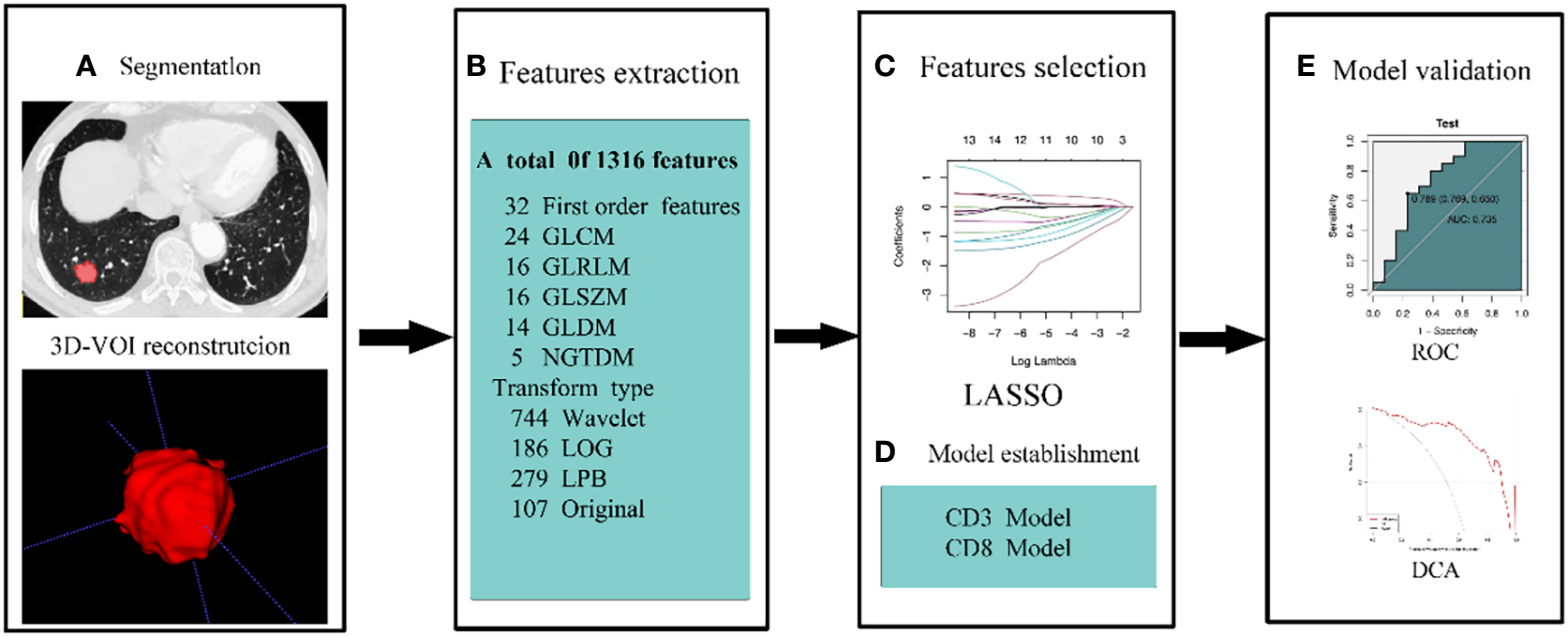
The workflow of our methodology. **(A)** To rebuild the 3D-VOI using ITK-snap, the ROI was manually formed on the lung window sequence of the CT images. **(B)** AK retrieved 1316 features from CT scans. Features were chosen by LOSSO and ANOVA-KW **(C)**. **(D, E)** ROC, calibration curve, and DCA were used to examine the diagnostic effectiveness of the model.

To determine the radiomics score (Rad-score) for each patient in order to build the radiomics model, the features acquired after LASSO regression were filtered and linearly fitted with the appropriate coefficient weights. The model underwent validation in the validation group. Analyze the sensitivity, specificity, and accuracy of the predictive model by describing receiver operating characteristic (ROC) curves and calculating the area under the curve (AUC). To assess the model’s calibration, plot a calibration curve. To determine the net benefit of the prediction model with various threshold probabilities, decision curve analysis (DCA) is utilized. Our method’s workflow is depicted in **Figure 2**.

### Statistical analysis

The K-S test for normal distribution and the homogeneity of variance test were carried out for quantitative data, and quantitative data that obeyed an essentially normal distribution were expressed as χ ± s. All data were analyzed using SPSS25 and R (version: 3.5.1, https://www.r-project.org). If not, they were written as M. (P25, P75). Independent samples t-test or Mann-Whitney U test was used to compare groups. The Fisher exact test or the X^2^ test were used to compare counts between groups. Count data were reported as frequencies and percentages. Statistics were judged significant at p< 0.05.

## Results

### Clinical features

This retrospective analysis comprised 105 NSCLC patients in total. High expression level and low expression level groups of patients were created based on the levels of CD3 and CD8 expression in the training and validation sets, respectively. In the training and validation sets, CD3 had a high expression rate of 61.1% and 60.6%, respectively, whereas CD8 had a high expression rate of 58.9% and 56.3%. Age, gender, maximal lesion diameter, histological type, tumor stage, and CD3 and CD8 expression levels were all subjected to univariate analysis. As shown in **Tables 2**, **3**, the findings revealed no significant differences in terms of gender, age, maximum lesion diameter, histological type, or tumor stage between the training cohort and validation cohort’s high and low CD3 and CD8 expression levels, and the differences were not statistically significant (all P values > 0.05).

**Table 2:** Relationship between clinicopathological characteristics and CD3 in patients with non-small cell lung cancer.

Characteristic	Training Cohort (n=72)		P	Validation Cohort (n=33)		P
	Low(n=28)	High (n=44)		Low (n=13)	Low (n=20)	
Age (years)	65.18 ± 8.78	65.84 ± 8.59	0.83	64.77 ± 8.88	64.05 ± 8.92	0.82
Tumer Size (mm)	24 (14.3,36.8)	22.5 (18,29.5)	0.88	21 (13.5,30)	18 (13.3,29.8)	0.97
Gender						
Male	17 (60.7%)	22 (50%)	0.37	7 (53.8%)	10 (50%)	0.83
Female	11 (39.3%)	22 (50%)	6 (46.2%)	10 (50%)		
Histology						
Squamous	7 (25%)	10 (22.7%)	0.83	1 (7.7%)	3 (15%)	1.00
Adenocarcinoma	21 (75%)	34 (77.3%)	10(76.9%)	15 (75%)		
Other	0	0	2 (15.4%)	2 (10%)		
Cancer Stage						
I	15 (53.6%)	26 (59.1%)	0.97	11 (84.6%)	14 (70%)	0.80
II	8 (28.6%)	7 (15.9%)	2 (15.4%)	5 (25%)		
III	3 (10.7%)	11 (25%)	0	1 (5%)		
IV	2 (7.1%)	0	0	0

**Table 3:** Relationship between clinicopathological features and CD8 in patients with non-small cell lung cancer.

Characteristic	Training Cohort (n=73)		P	Validation Cohort (n=32)		P
	Low (n=30)	High (n=43)		Low (n=14)	High (n=18)	
Age (years)	64.77 ± 8.44	64.90 ± 9.22	0.99	65.57 ± 8.75	65.78 ± 7.98	0.63
Tumer Size (mm)	22.5 (14.5,29.3)	22 (16,29)	0.92	18 (13,31.5)	26 (18.5,32.7)	0.97
Gender						
Male	14 (46.7%)	22 (51.2%)	0.71	9 (64.3%)	11 (61.1%)	0.86
Female	16 (53.3%)	21 (48.8%)	5 (35.7%)	7(38.9%)		
Histology						
Squamous	5 (16.7%)	11 (25.6%)	0.79	2 (14.3%)	4 (22.2%)	0.73
Adenocarcinoma	24 (80%%)	31 (72.1%)	10 (71.4%)	13(72.2%)		
Other	1 (3.3%)	1 (2.3%)	2 (14.3%)	1(5.6%)		
Cancer Stage						
I	20 (66.7%)	29 (67.4%)	0.17	9 (64.3%)	8 (44.4%)	0.47
II	7 (23.3%)	8 (18.6%)	3 (21.4%)	4 (22.2%)		
III	1 (3.3%)	6 (14%)	2 (14.3)	6 (33.4%)		
IV	2 (6.7%)	0	0	0		

### Texture feature extraction and selection

**Figure 3:**
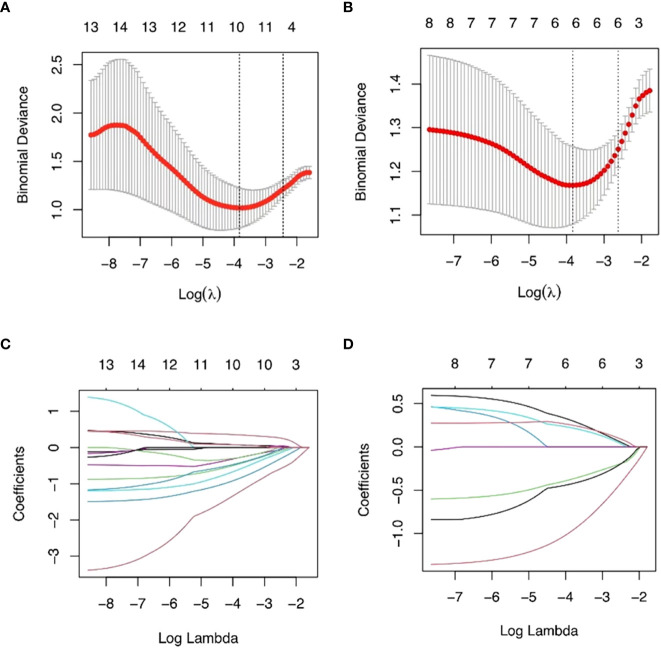
The most advantageous subset of radiomics characteristics was extracted using the LASSO technique and 10-fold cross-validation. The best feature selection based on AUC values is shown in **(A, B)**. The best value that yields the lowest binomial deviation is shown by the vertical dashed line’s log() value. When the ln () value rises to this level, the AUC hits a peak matching to the ideal number of radiomics characteristics. **(C, D)** Radiomics portion LASSO coefficient distribution.

**Figure 4:**
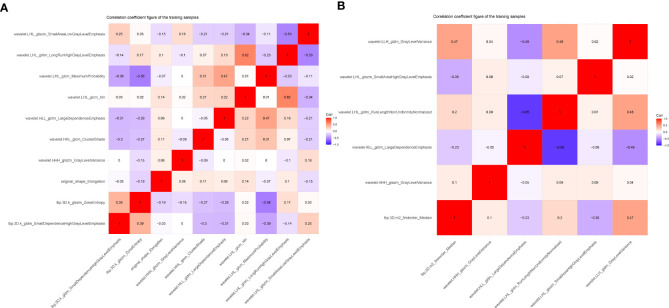
Heat map showing correlations for identifying histological features in CD3 **(A)** and CD8 **(B)** expression imaging. The values of the color bars on the right are the correlation coefficients, and hues imply strong and positive correlations while light colors suggest negative correlations.

**Figure 5:**
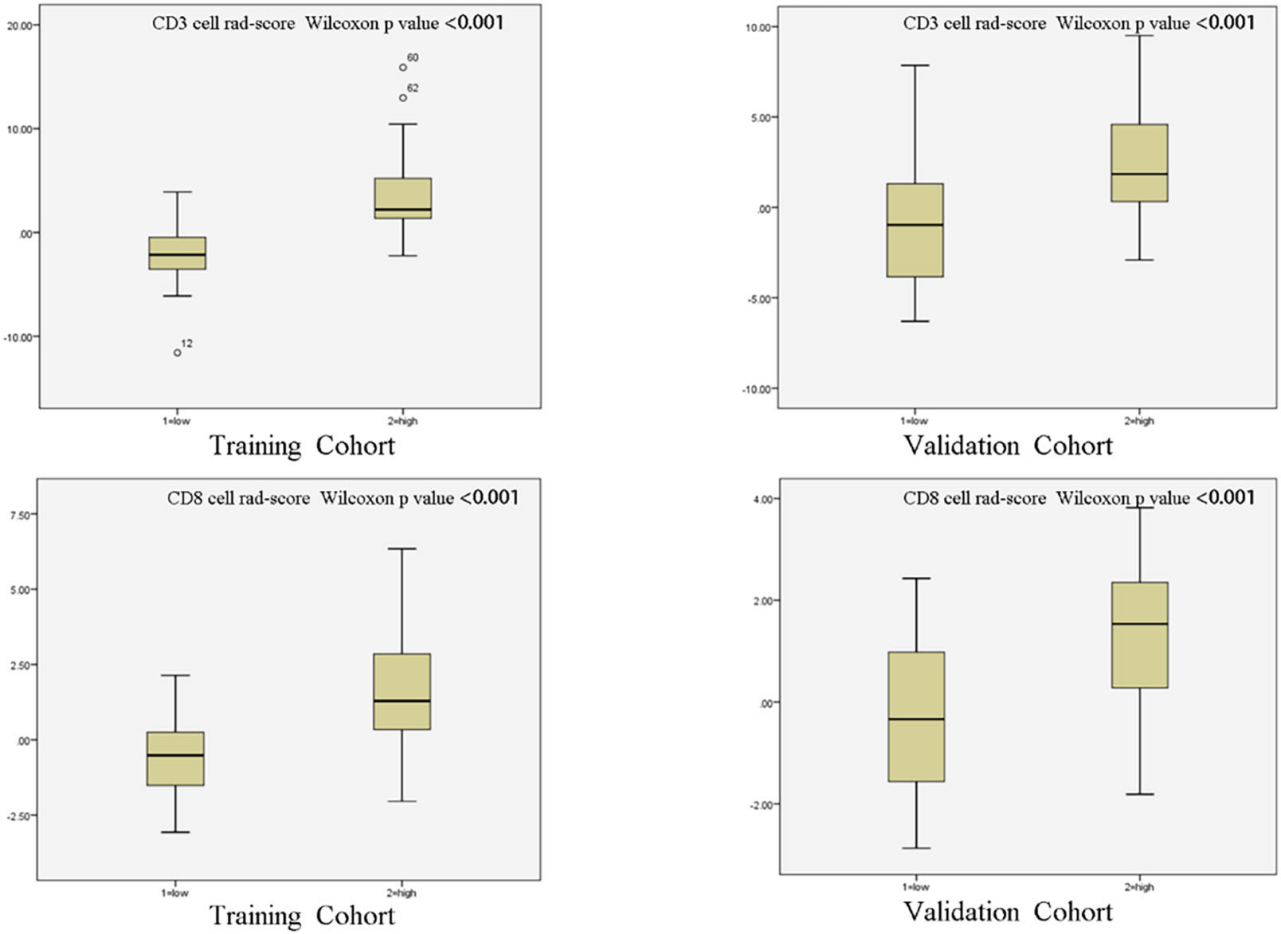
Radiomic scores of patients in different cohorts. The radiomic scores of patients with strong infiltration of CD3 and CD8 cells were substantially higher than those of patients with low infiltration in both the training cohort and validation cohort.

**Figure 6:**
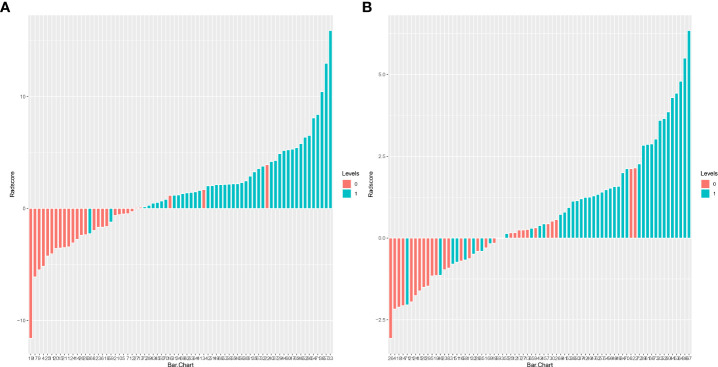
Adiomics score (Rad-score) bar graph for the CD3 **(A)** and CD8 **(B)** training set. The Rad-score value is shown on the Y-axis; positive values indicate high expression forecasts, negative values indicate low expression predictions, red bars indicate true soft expression cases, and blue bars indicate real high expression cases. Correct predictions are represented by red bars with negative values and blue bars with positive values, whereas wrong predictions are blue bars with negative values and red bars with positive values.

The training set’s CD3 and CD8 models have 10 and 6 optimum features, respectively, after the LASSO algorithm’s feature reduction and 10-fold cross-validation (**Figure 3**). The Spearman correlation heat map between radiomics features under each modality is shown in **Figure 4**. The rad-score of the high-expression group was higher than that of the low-expression group in the CD3 and CD8 training and validation sets, respectively (P<0.05) (**Figure 5**). The differences between 10 features in CD3 and 6 features in CD8 in the training set were statistically significant (P<0.05) (**Tables 4**, **5**). Each patient’s rad score in the training set was shown on a bar graph (**Figure 6**).

**Table 4:** Texture characteristics of patients in training cohort of CD3 cells infiltrating group.

Variables	Low (n=28)	High (n=44)	P value
lbp.3D.k_gldm_SmallDependenceHighGrayLevelEmphasis	-0.52 (-0.89,0.12)	0.20 ± 1.04	0.02
lbp.3D.k_glszm_ZoneEntropy	-0.40 ± 1.06	0.26 ± 0.87	0.01
original_shape_Elongation	0.35 ± 0.83	-0.22 ± 1.04	0.17
wavelet.HHH_glszm_GrayLevelVariance	0.57 (0.20,0.87)	-0.16 (-0.82,0.65)	0.01
wavelet.HHL_glcm_ClusterShade	0.09 (-0.29,0.66)	-0.24 ± 0.59	0.03
wavelet.HLL_gldm_LargeDependenceEmphasis	0.34 ± 1.16	-0.23 (-0.71,0.31)	0.04
wavelet.LHL_glcm_Idn	0.52 (0.07,1.13)	-0.14 (-0.37,0.19)	0.00
wavelet.LHL_glcm_MaximumProbability	-0.13 (-0.49,1.11)	-0.41 (-0.71,0.08)	0.04
wavelet.LHL_glrlm_LongRunHighGrayLevelEmphasis	0.39 ± 0.99	-0.31 (-0.60,-0.13)	0.01
wavelet.LHL_glszm_SmallAreaLowGrayLevelEmphasis	-0.32 ± 0.96	0.20 ± 0.98	0.03

**Table 5:** Texture characteristics of patients in training cohort of CD8 cells infiltrating group.

Variables	Low (n=30)	High (n=43)	P value
lbp.3D.m2_firstorder_Median	-0.29 ± 0.69	-0.04(-0.44,0.46)	0.03
wavelet.HHH_glszm_GrayLevelVariance	0.62(0.35,0.77)	-0.26(-0.26,0.74)	0.01
wavelet.LHL_glrlm_RunLengthNonUniformityNormalized	-0.31 ± 0.99	0.22 ± 0.96	0.03
wavelet.LHL_glszm_SmallAreaHighGrayLevelEmphasis	0.33 ± 1.23	-0.23 ± 0.73	0.03
wavelet.HLL_gldm_LargeDependenceEmphasis	-0.31 ± 0.64	0.05(-0.64,0.94)	0.04
wavelet.LLH_gldm_GrayLevelVariance	0.10(-0.32,0.79)	-0.16(-0.68,0.36)	0.01

### Model development and verification

**Figure 7:**
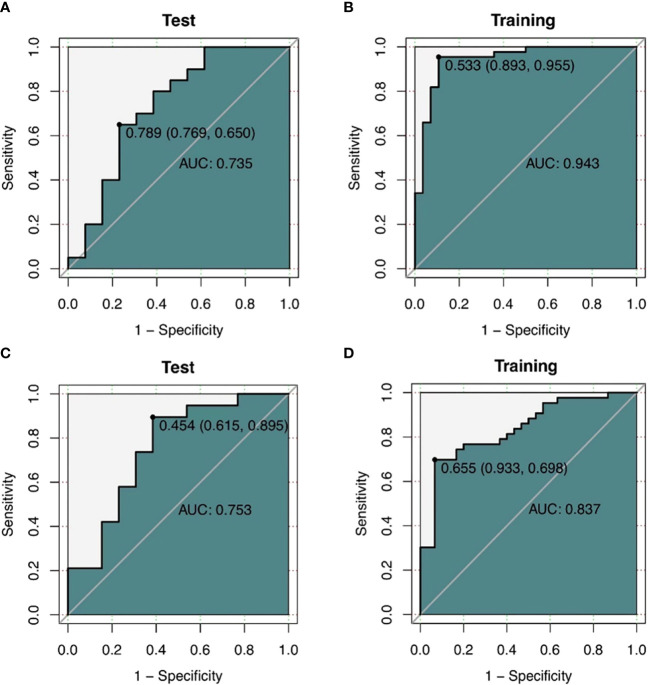
**(A, B)** ROC curves of the CT texture feature model predicting CD3 expression. a ROC curve of the training cohort (n=72). **(B)** shows ROC curves of the validation cohort (n=33) **(C, D)** ROC curves of CD8 expression predicted by CT texture feature model. ROC curves of the training cohort (n=73). **(B)** shows ROC curves of the validation cohort (n=32).

Two logistic regression models were created in this work to predict the levels of CD3 and CD8 expression, and the associated ROC curves were displayed and examined. Results indicated that the CD3 model’s AUC was 0.943 (95% CI 0.886-1), with sensitivity, specificity, and accuracy values of 0.955, 0.893, and 0.931, respectively. When the model was applied to the validation set, the AUC was 0.735 (95% CI 0.541-0.929), with sensitivity, specificity, and accuracy values of 0.65, 0.769, and 0.697, respectively (**Figures 7A, B**). The logistic regression model for CD8 thus achieved an AUC of 0.837 (95% CI 0.745-0.930) in the training set, with sensitivities, specificities, and accuracies of 0.698, 0.933, and 0.795, respectively. With sensitivities, specificities, and exactness of 0.895, 0.615, and 0.781, respectively, the analysis in the validation set gave an AUC of 0.753 (95% CI 0.568-0.938). (**Figures 7C, D**).

**Figure 8:**
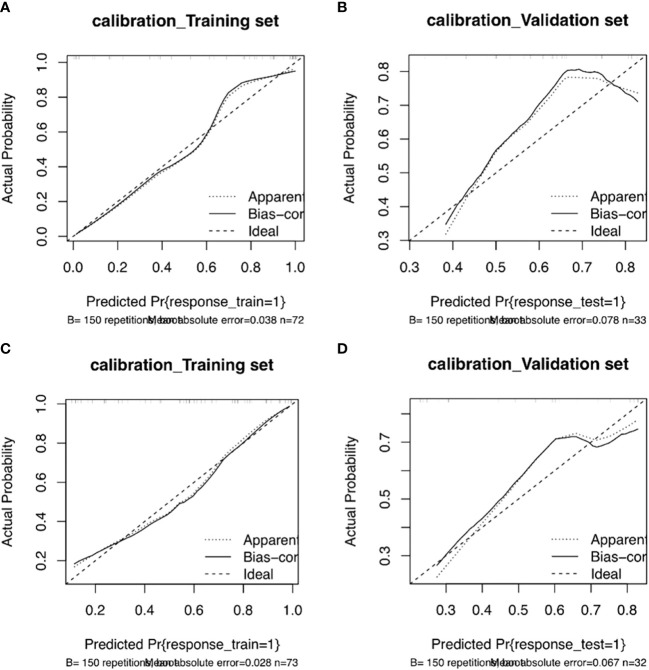
Column line plot calibration curves for the CD3 training set **(A)** and validation set **(B)**. The incidence of projected CD3 expression is represented by the horizontal axis. The incidence of detected CD3 expression makes up the vertical axis. The reference line, which is red on the diagonal, shows that the predicted value and the actual value are same. The fact that the forecasts mainly coincide with the diagonal line shows that they are correct. Column line plot calibration curves for the CD8 training set **(C)** and validation set **(D)**. The projected incidence of CD8 expression is represented on the horizontal axis. The incidence of detected CD8 expression makes up the vertical axis. The reference line, which is red on the diagonal, shows that the anticipated value and the actual value are same. The fact that the expected outcomes generally coincide with the diagonal line shows that the predictions were correct.

**Figure 9:**
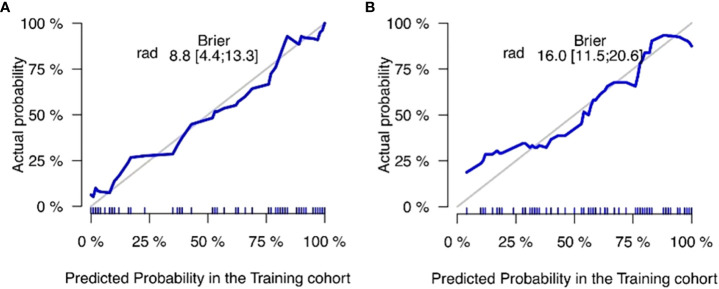
Non-small cell lung cancer (NSCLC) CD3 **(A)** and CD8 **(B)** expression is predicted using a decision curve analysis, with the horizontal axis displaying the range of risk thresholds and the vertical axis displaying the net benefit. The gray line in the illustration denotes the presumption that all lesions are positively expressed, and the more the blue curve deviates from the gray line, the greater the net advantage of the model.

The training and validation sets’ calibration curves for both models revealed that the projected probability in the training and validation sets closely matched the levels of actual gene expression, demonstrating the models’ excellent calibration (**Figure 8**). The decision curves for the radiomics model revealed that utilizing the CT-based imaging omics label to predict CD3 and CD8 expression had a net gain higher than categorizing all patients equally as positive or negative in the training set (**Figure 9**).

## Discussion

Based on the radiomics properties of CT images, our study examined the expression of CD3 and CD8 in non-small cell lung cancer. A total of 1316 radiomics features were extracted from the pretreatment images, and 16 optimal features were ultimately chosen by LASSO regression. These features included first-order features (1), high-order GLDM (2), GLRLM (1), and GLSZM (2) texture features for predicting CD8 expression, as well as morphological features that predicted CD3 expression and higher-order GLDM (2), GLCM (3), GLRLM (1), and GLSZM (3) texture features. In both the training and test groups, the model’s AUC values, sensitivity, specificity, and accuracy were increased, indicating that the CT-based radiomics model has superior predictive performance. The test group’s AUC values (0.735 and 0.753) were higher than those of the CD3 and CD8 training groups (0.943 and 0.837, respectively), indicating strong model generalization. There was a statistically significant difference between this feature in the high and low expression groups of CD3, indicating that different levels of imaging features might have the potential to distinguish between different expressions. This difference was found in the shape feature Elongation (elongation rate), which indicates the relationship between the two largest major components of ROI shape. The ROI’s voxel grayscale value distribution is primarily described by the first-order features, where the median value denotes the ROI’s median grayscale value. The findings of the current investigation revealed that the median value was statistically different in reflecting the level of CD8 expression (P<0.05), and it has been demonstrated that for CD8, the median value is often utilized for the categorization of high or low expression (18).

The heterogeneity of the tumor is highlighted by GLDM, which is based on voxel values assessing the differences between neighboring voxels and describes the correlation of grayscale in pictures with considerable dependency. According to changes in growth factor activity, angiogenesis, and the tumor microenvironment, local tumor cell proliferation or death, variations in metabolic activity, and enhanced or reduced angiogenesis may result from tumor heterogeneity (19). According to research, there is a significant association between GLDM and CD8, which is similar with the findings of the current study (20). Within the tumor, CD8 is unevenly distributed, with both T-cell infiltrated and T-cell excluded areas present. A diverse pattern of tumor metabolism may result from the variable distribution of CD8. Larger LRHGLE values indicate higher gray values, more clustered high gray values, relatively long run lengths, and a coarser texture structure. This may reflect the correlation of highly expressed intratumor regions with high grayscale on the image. GLRLM describes the arrangement of pixels with the same grayscale in a specified direction. Our trait was statistically significant in this investigation for forecasting CD3 expression (p ≤0.01). A higher RLNN number denotes a more homogeneous distribution of image tour lengths. GLSZM represents the distribution of uniform voxels in the 3D area similarly to GLRLM (21). Higher values suggest greater variation between gray values and greater heterogeneity of image texture. GLV measures the variance of gray values in the region. The tumor’s general texture distribution is not uniform, which indicates that its heterogeneity is substantial. This feature is more prominent in the high expression group than in the low expression group, which may be explained by the idea that the high expression cells’ nuclei are larger, denser, and more likely to exhibit abnormal differentiation. Additionally, the single voxels that represent the cell population may have higher gray values, which would result in a relatively high overall pixel gray value, giving the feature a high and low uneven triad appearance. ZE denotes the degree of unpredictability and chaos in the regional distribution, while SALGLE measures the proportion of the joint distribution of smaller-size areas with lower gray values in the image and SAHGLE denotes the proportion of smaller-area regions with higher gray values in the image.

Numerous studies have shown that the existence of CD3 and CD8 subpopulations of tumor-infiltrating lymphocytes is consistently linked with prognosis and survival in patients with non-small cell lung cancer (22–24). Noteworthy, the host immune system and the lymphocyte profile, prognostically essential in solid tumors, can be impacted by oncologic treatments. As an example, radiation-induced lymphopenia has been clearly linked with poorer survival outcomes in cancer patients, including NSCLC (Grossman et al., 2015; Venkatesulu et al., 2018; Jin et al., 2017; Ladbury et al., 2019; Tang et al., 2014) (25–29).Lining Li et alfindings ‘s supported the notion that CD8 T cells are reliable indicators for gauging patient response and survival after ICI (30). Another work by Hyojin Kim et al. demonstrated the significance of CD3 and CD8 T cells subsets in adaptive immunity and their value as predictive biomarkers of PD-1 inhibition in non-small cell lung cancer (9). The current work is significant because it uses CT scans of tumors to predict the expression of CD3 and CD8.

When compared to peripheral blood, TIL had considerably larger concentrations of CD8 and CD3 T cells. In this investigation, the researchers discovered that cancerous stromal tissue and cancer nests both included CD3 T cells and CD8 T cells. Patients’ 5-year prognoses were markedly improved when there were high concentrations of CD3 T cells in the tumor sites. High amounts of CD8 T cells, on the other hand, were linked to a bad prognosis. Increased apoptosis was linked to high CD3 T cells counts in NSCLC patients (31). Therefore, it is important to understand how CD3 and CD8 T cells contribute to tumor immunity.

Currently, postoperative pathological immunohistochemistry analysis is mostly used to identify tumor-infiltrating cells. It is extremely important for patient prognosis if the CD3 and CD8 expression of patients with non-small cell carcinoma can be resolved with preoperative non-invasive testing. Radiomics analyzes tumor phenotypic variations, extracts numerous quantitative variables from radiographic images, and offers non-invasive prognostic data. Parts of the applicability to non-small cell lung cancer have been documented in earlier research. In order to build an imaging-omics model to forecast non-small cell lung Ki67 expression to gauge patient survival, Yao et al. integrated clinical data (32). In ESCC patients, Wen et al. found a significant relationship between pretreatment CT radiomics characteristics and immunological biomarker expression status (33). Combining radiomics characteristics and clinical factors can enhance the CD8 expression’s prediction effectiveness.

Previous research has shown a correlation between the levels of CD3 and CD8 expression and various clinicopathological characteristics, including age, gender, lung cancer histological subtype, tumor size, and clinical stage (26). In contrast to the findings of earlier studies, which demonstrated that patients with advanced stages of cancer are more likely to have high levels of CD3 and CD8 expression, this group of cases’ differences between the high and low expression groups of CD3 and CD8, respectively, in tumor stage were not statistically significant (P > 0.05) (34).Because there were fewer advanced cases in this research, early-stage cases were more common. In accordance with the findings of earlier studies, our study found no statistically significant differences between the high and low expression levels of CD3 and CD8 in either the training or validation groups in terms of patient age, gender, tumor size, or histological lung cancer staging (P > 0.05).

We have put texture correlation models into practice and shown their usefulness in medical settings. Texture analysis is more accurate than clinical models, with the former having somewhat superior calibration performance and discriminative power, with AUCs in the training cohort of 0.943 and 0.837, respectively. Research has demonstrated that radiomics may accurately predict CD3 and CD8 expression in a variety of tumor types (18, 35, 36). In this work, we used CT-based radiomics characteristics to create a model to forecast CD3 and CD8 expression in non-small cell lung cancer. For 10 and 6 radiomics characteristics, the findings revealed a significant difference between the negative CD3 and CD8 group and the positive CD3 and CD8 group (p <0.05).

## Limitations of our study

Our research has certain drawbacks. First off, this study’s tiny sample size may raise more questions regarding selection bias. Additionally, this study was a retrospective single-center investigation. There is a need for more research involving many patients and several centers. Second, only CD3 and CD8 were used for this study; more research is required on other biological markers of tumor cells, such as CD4 and FOXP3. Third, additional rather uncommon pathological forms of non-small cell lung cancer were not included in this study. The effectiveness and generalizability of imaging histology will thus be enhanced in future investigations by increasing the sample size to include more lung cancer patients with additional pathological kinds and adding more clinical data. Powerful modeling tools are now available to mine the vast quantity of image data that is currently accessible and show the underlying intricate biological pathways thanks to the advent of quantitative imaging techniques and machine learning (19). To create models with the best prediction performance, more sophisticated radiomics techniques like machine learning and deep learning should be built.

## Conclusion

Conclusion: In patients with non-small cell lung cancer, our study shows a significant association between CT imaging histological characteristics and immune biomarker expression status. The expression of CD3 and CD8 in NSCLC may be predicted using genomics-based CT scan pictures, which may have some therapeutic and societal utility as a new non-invasive method to comprehend the molecular information of NSCLC cells.

## Data availability statement

The original contributions presented in the study are included in the article/**Supplementary Material**. Further inquiries can be directed to the corresponding authors.

## Ethics statement

Ethical review and approval was not required for the study on human participants in accordance with the local legislation and institutional requirements. Written informed consent from the participants was not required to participate in this study in accordance with the national legislation and the institutional requirements.

## Author contributions

LujC and MY conceived and designed this study. LujC conducted the study and collected important background data. LulC, LS and HN helped to collect the clinical data. LujC drafted the manuscript. JW performed the histological examination. YX performed R statistical analysis. JY, MY and ZZ put forward many opinions on the manuscript. All authors read and approved the final manuscript. All authors contributed to the article and approved the submitted version. 
