# Supplementary material for: Prediction of CD3 T cells and CD8 T cells expression levels in non-small cell lung cancer based on radiomic features of CT images
Source: Front Oncol. 2023 Feb 13;13:1104316. doi: 10.3389/fonc.2023.1104316 (PMC9968855; doi:10.3389/fonc.2023.1104316)
Supplement: Supplementary file 1 [file DataSheet_1.pdf]

data_Texture characteristics of patients in training cohort of CD3 cell

	Label	lbp.3D.k_gldm_SmallDependenceHighGrayLevelEmphasis
23	0	-0.924282739
33	0	-0.422436942
93	0	-0.150897089
6	0	0.071880339
4	0	-0.994065965
94	0	-1.925887776
90	0	0.211332302
83	0	-0.514336176
74	0	-0.898139661
97	0	2.495662001
53	0	-0.486665151
17	0	0.129069198
42	0	-0.959750373
55	0	0.540017401
1	0	-0.238188631
100	0	1.34182125
16	0	-1.036728122
98	0	0.583914945
45	0	-0.966925337
52	0	-0.709924658
39	0	-0.604304026
48	0	0.176529929
30	0	-0.007334164
66	0	-0.784667833
84	0	-0.845061081
29	0	-0.530613125
99	0	-0.629722077
80	0	-0.874085593
67	1	-0.219144395
3	1	1.119603931
35	1	-0.514563654
78	1	-0.791063047
70	1	-0.363026389
24	1	-0.73675753
54	1	0.970835725
58	1	-0.637367126
22	1	1.400874844
92	1	0.379358048
34	1	0.774314296
68	1	0.301528313
47	1	-0.98081581
50	1	-0.711389772
51	1	0.830043835
31	1	0.897261424
32	1	1.188163969
71	1	-0.922832413
56	1	-0.597065549
21	1	3.640936274
103	1	1.95429976
64	1	-1.379023605
89	1	1.07526622

37	1	-0.412913462
18	1	-0.008043789
38	1	0.215715038
102	1	1.542751787
62	1	0.079146825
73	1	0.82504436
79	1	1.054999
27	1	0.544945457
8	1	1.566074968
60	1	-1.443798839
87	1	-0.052312467
25	1	0.857291183
101	1	0.067373506
49	1	-0.317795734
44	1	-1.24800673
61	1	0.836963175
41	1	-1.115428039
43	1	0.398102023
28	1	0.578565666
81	1	-0.448264018
69	1	-1.246058106

lbp.3D.k_glszm_ZoneEntropy
-1.214152352
0.462413614
-0.783950331
0.420777042
-1.43169633
-2.744543132
-0.930868691
-0.409294661
-0.651598583
0.146136954
-0.893902504
1.322909728
-0.123049821
-0.004995204
0.269679925
2.676212743
-0.878621638
1.089846527
-0.600094189
0.369787558
-0.629323843
-0.415068908
-1.173077298
-0.821211154
-0.170337117
-0.837480501
-1.434066049
-2.017631369
1.180919592
-0.539557732
0.295891209
-1.695032525
-0.181341574
-0.40917306
0.066439762
-0.749573355
1.485846191
0.903853087
1.296090649
1.580843788
0.049544814
0.950233695
0.126817576
0.81571783
-1.111004759
-0.178313425
0.758544173
-1.201993521
1.462971215
0.109577877
0.499287827

0.566459678
0.661912695
0.392372668
0.068643769
1.231368142
0.896753483
0.729499905
-0.827575967
1.003530769
1.350161837
-0.745808447
0.645739418
-0.084171542
0.176273594
-1.159147842
0.774195636
-1.168612557
0.416294953
1.023067029
1.196092664
-1.256439639

original_shape_Elongation
1.302217912
0.559319335
-0.870108768
0.770831304
0.560989493
0.764465673
2.131210946
-0.473809189
1.049546332
1.315292575
0.641448983
1.286338868
-0.550947179
-0.600413719
0.123461133
0.911401879
0.666649673
0.245102385
0.952047213
-1.084840947
-0.117808963
0.140481749
0.933117712
-1.329096778
0.891879042
-0.394864678
0.516516993
-0.579403141
-0.379585181
-0.817810028
1.018090957
-2.78993126
-1.51670539
0.874053326
-0.120625191
0.86714745
-0.477706795
-0.556039183
-0.390614921
-1.108049904
0.956413395
-0.563774741
0.863555437
-1.724209871
0.050753683
1.733655945
-1.238965006
-0.56793104
0.603760856
-1.586617148
-1.048787995

-0.222133983
-0.062554443
-0.34625803
-0.117022935
0.307454036
0.278604759
-0.939503525
1.195156579
0.597669437
-1.77402434
1.043693256
0.923644588
-0.601112509
-1.718292928
-0.507683543
-0.727357281
1.300230085
-0.764250622
-0.933502956
-0.701957179
1.928098301

wavelet.HHH_glszm_GrayLevelVariance
0.872608828
0.442691308
0.739918224
0.494751654
0.442691308
-0.321606515
0.765129448
0.872608828
0.653263146
0.442691308
-0.321606515
0.872608828
0.872608828
0.653263146
0.872608828
-0.321606515
0.442691308
0.872608828
0.872608828
0.739918224
0.872608828
-1.814375671
0.872608828
0.200862703
0.442691308
-0.321606515
-0.321606515
0.442691308
-0.321606515
0.653263146
0.300233452
0.200862703
0.872608828
-1.468053205
0.442691308
0.442691308
-0.321606515
-0.321606515
-0.321606515
0.872608828
0.835418727
-2.99664885
0.442691308
-0.847061251
-1.101502217
-1.814375671
-1.101502217
-0.321606515
-0.321606515
0.653263146
-0.321606515

0.442691308
0.783782881
0.872608828
-0.321606515
0.200862703
-0.321606515
0.872608828
0.872608828
-1.814375671
-1.814375671
-0.717322849
0.835418727
0.872608828
-1.101502217
0.442691308
-0.004773854
-2.99664885
-0.321606515
0.200862703
-3.325804451
0.872608828

wavelet.HHL_glcm_ClusterShade
0.354373899
0.0286393
-0.068785539
-0.173353514
0.0018536
3.442523861
-0.316433679
-1.049179939
0.433251279
0.12158051
0.803412481
-0.883071412
-0.111697436
0.168806234
-0.228135219
0.183011966
-0.7926562
-1.067852406
5.164925391
0.237488397
1.225947857
-0.653732484
0.058828347
0.726705256
0.120658042
2.392431427
-0.502645786
1.082866167
-0.46131682
0.235314645
-0.154619033
-0.398051326
-0.563335583
0.046293063
-0.607455059
0.092767537
-0.073384694
-1.642024214
0.668284139
-1.03718981
-0.29655919
0.036744468
-0.544440454
0.026399066
0.096910908
-0.169865812
0.05344372
-1.809359829
-0.253648383
-0.674738261
-0.151065938

-0.334592271
-0.425356569
-0.617064034
0.280550479
-0.17280836
-0.538847989
-0.596089427
-0.107265358
0.416721502
0.323866392
-0.401602242
-0.144868484
-0.591609831
0.733427702
-1.314268554
-0.4689977
0.439591506
1.533502503
-0.900483441
0.177092539
-0.409761903

wavelet.HLL_gldm_LargeDependenceEmphasis
1.305717954
0.188423784
0.108955678
0.811916488
0.559987454
0.819657698
-0.696264172
1.898708155
-0.751057049
-1.566031524
2.065363337
1.45516516
0.555323356
0.499014431
-0.16842493
-1.577706888
3.827850161
-0.173484891
-0.639213689
-0.08499422
-0.972336534
0.427864064
-0.317007839
1.739022034
-0.492828754
-0.008825159
0.371511502
0.316204748
-0.208292431
-0.142651803
-0.720736952
-0.821544383
-2.995182506
1.117077412
-0.763792926
0.104764184
-0.384538191
-0.392901234
-0.913357055
-0.618289209
-0.246656115
-0.200731795
0.599422851
0.341684854
-0.043555438
-0.60148753
0.339999907
-0.527015821
-0.902136886
0.928789206
1.057986197

-0.609412037
0.233488987
-1.812827929
-0.044562671
-0.00957377
-0.030728509
-0.253493584
0.963160322
-0.899027719
0.692911712
0.139616406
-0.308324381
0.201469149
-1.601234836
1.136031896
0.554107383
1.034367811
-0.70893219
-0.660730291
-0.746198449
-0.779471991

wavelet.LHL_glcm_Idn
-0.113184943
0.271479232
-0.175502209
1.404293823
-0.233216142
-1.567300627
-0.159040616
0.375051509
1.432166595
0.892721511
0.728263531
0.063434225
-0.306262526
0.766571201
1.158629628
0.603981947
1.028145415
0.982273026
1.165544255
1.432191193
1.274900554
0.089211615
0.214100323
0.734272111
1.193252979
0.385460919
0.451264514
0.349804527
-0.517699793
-0.365659909
-0.10486405
0.017408847
-4.303536243
-0.191052033
-0.142424445
0.512778509
-0.055501327
-1.844201761
0.834090979
0.076827904
-0.699364734
0.3684311
0.066727777
-0.193359643
0.642436632
0.381614014
-0.440073468
-0.128130884
0.711150067
0.300394261
0.239564172

-1.501521884
-0.352250149
-1.616759516
0.056697692
-0.135366426
-3.70543711
0.188553148
-0.141831049
-0.261927589
0.481745788
-0.006565092
-0.281393154
0.022208961
-0.21591416
-2.205772812
-0.189744541
-0.163822171
0.188144203
0.981714397
-0.247683795
-0.501138284

wavelet.LHL_glcm_MaximumProbability
-0.192853873
0.257610323
-0.101271502
-0.981862206
-0.37221409
2.792134373
-0.020650047
2.765153035
-0.121877065
-0.188963892
2.591067097
-0.51049982
-0.293621792
-0.844885421
-0.923622301
-1.353575594
2.027300866
-0.220036698
1.879702232
-0.417010086
-0.128637726
0.139281235
1.337563846
1.042615446
-0.654897812
1.133685095
-0.559386723
0.890856774
-0.72962638
-0.373351262
-0.428954126
0.809038202
-0.435995809
-0.377864012
-1.15711963
1.102681199
-0.714792611
-0.352200903
-1.485329803
-0.313457583
-0.735848709
-0.075665839
0.08525181
-0.579909171
1.502578685
-0.704459526
-0.462812236
0.576740071
-0.836536934
1.187455308
-0.256153443

-0.269590589
-0.84379605
-0.680438747
-0.500093644
-0.26614552
-0.443781253
-0.238820154
-0.633686407
-0.980959335
1.579582057
0.444215531
-0.856156791
-0.130245621
0.33453382
2.48398825
-0.78332046
0.037212033
0.158730179
-1.545624875
-0.492251276
-0.588122119

wavelet.LHL_glrlm_LongRunHighGrayLevelEmphasis
0.682428076
-0.089060545
-0.511451007
2.276572834
0.182825822
-1.623093343
-0.457149961
0.119899057
1.178241586
-0.675676179
-0.612729458
-0.097510579
1.007081808
2.054269724
1.81392276
1.173050413
0.844253729
1.401102532
0.897476368
1.554001332
-0.626177364
-0.191056562
-0.670248449
0.521923529
1.68180768
-0.309170532
-0.105580019
-0.291457295
-0.115480961
-0.654923417
-0.165597591
-0.492868493
-1.675271615
0.877332291
-0.315744226
-0.193005217
-0.542049559
-1.336979941
1.025578688
-0.785922859
-0.028967439
4.144718162
-0.429372312
-0.267182901
-0.39344842
-0.287719351
-0.260126454
-0.801287004
-0.34092916
-0.165838388
0.019395123

-1.245621632
-0.10037343
-1.571517536
-0.22037444
-0.394814834
-1.478628679
-0.319731644
0.007225625
-0.263235107
-0.03183184
-0.192678668
-0.454035372
-0.329268153
-0.621873687
-1.463617428
-0.216011416
0.626212697
-0.669581538
1.636598156
-0.469892004
-0.16975398

wavelet.LHL_glszm_SmallAreaLowGrayLevelEmphasis
-0.683259695
-0.130307138
1.954000507
-1.512574358
0.047536619
-2.26354336
0.23771802
-0.252427987
-1.293895643
0.519218076
0.243230385
0.654345173
0.220893165
-0.902177382
-1.221194742
-1.506705206
0.142405167
-0.802360617
-1.310413069
-0.969824007
0.939472328
-0.172632562
0.121139503
-0.822930293
-1.437190583
1.206403103
-0.433735803
0.481637364
0.888520508
0.512551171
0.121714003
0.828365976
2.126700788
-0.876456688
-0.305114878
0.511998261
0.587912029
-1.293445984
-0.848823927
1.970230649
0.548039096
-1.437694213
-0.048510102
0.24851462
-0.622550836
0.106781798
0.624051832
0.765178132
1.452681934
-0.120043222
-0.213333548

-1.425118738
0.391828738
2.108107912
1.686359469
1.541565037
1.832164781
-0.452977071
0.219361518
-0.733348048
-1.622351559
0.288711922
-0.253592746
0.862019504
0.204874797
-0.781660441
0.173127637
-0.530892398
0.893226266
-1.359947996
0.043529565
0.334917492

Radscore_train
-3.418182697
-0.61854548
1.684741827
-5.154760284
-0.487603525
-1.665124277
-0.455864312
-1.954395009
-5.472260285
-0.546419397
-4.041464337
-0.266490619
0.109936327
-2.74914189
-3.530285139
1.16668642
-6.109198337
-1.603161775
-11.59252122
-3.564783628
-3.486636262
3.903117717
-1.672801207
-3.066604771
-4.242481213
-2.39021764
0.090140853
-2.336432221
4.896035124
2.462314896
1.182933088
3.775072538
15.88965596
1.207741467
3.273729814
-2.250026988
4.282646897
10.43014328
0.285576147
4.194290251
1.610801936
2.019509768
0.474728616
5.170541467
0.541272389
2.204903118
5.300156873
5.792560256
2.12387189
0.671660182
2.033370895

6.364611253
2.1473673
8.091985467
2.202107129
2.310672531
12.96055219
2.172289645
-1.204644495
5.236901425
2.891420634
1.397122246
1.434653516
1.482966432
5.425347317
6.522455753
3.560664239
2.128282357
1.321481837
0.812132039
8.395576621
0.153496891
