# Supplementary material for: Prediction of CD3 T cells and CD8 T cells expression levels in non-small cell lung cancer based on radiomic features of CT images
Source: Front Oncol. 2023 Feb 13;13:1104316. doi: 10.3389/fonc.2023.1104316 (PMC9968855; doi:10.3389/fonc.2023.1104316)
Supplement: Supplementary file 2 [file DataSheet_2.pdf]

data_Texture characteristics of patients in training cohort of CD8 cell

	Label	lbp.3D.m2_firstorder_Median
30	0	-1.06031778
36	0	-0.22019396
65	0	-0.984210428
72	0	-0.343233984
57	0	-0.676610659
73	0	0.671096336
20	0	-0.648611477
82	0	-1.08023115
93	0	0.907341287
4	0	-0.566970827
53	0	-0.992804416
90	0	-0.558428608
9	0	-0.073242865
11	0	-0.668379911
17	0	0.448784951
39	0	-1.117441546
12	0	1.163164096
74	0	-1.413326596
80	0	0.170118619
1	0	-0.12041401
26	0	-1.071109902
48	0	-0.020395829
66	0	-0.213242676
55	0	-0.418655728
5	0	0.93952609
16	0	-0.761223505
99	0	0.482185123
29	0	-0.912216542
6	0	0.369677704
91	0	-0.067801263
95	1	-0.103295602
28	1	-0.88262521
104	1	-0.625317818
54	1	0.71172902
58	1	0.447954292
88	1	4.106965221
68	1	0.366381253
102	1	0.496373847
43	1	-0.30562455
61	1	0.488428913
67	1	0.021986825
22	1	-0.886603599
63	1	-0.16454075
19	1	-0.040349997
13	1	-0.951158125
27	1	0.094259611
34	1	-0.061595143
31	1	0.203761893
75	1	-0.653016673
62	1	0.219941519
69	1	-0.590012484

8	1	-0.435954627
76	1	3.284482968
24	1	-0.157246569
51	1	0.457440879
96	1	0.198764641
21	1	-0.936460254
46	1	-0.343233984
101	1	-0.113988272
38	1	3.026643174
100	1	0.205791835
40	1	-0.284243668
89	1	1.376738357
81	1	-0.651961268
44	1	1.663095508
49	1	-0.376866802
15	1	0.198497754
77	1	0.909626117
78	1	-1.637255944
25	1	1.428011213
23	1	-0.956025212
70	1	0.298526577
41	1	-0.210855409

firstorder一阶特征	Median(中值)
	Gray Level Variance(GLV,灰度方差)?
	?Large Dependence Emphasis(LDE,大依赖强调)
	Run Length Non-Uniformity Normalized（RLNN，归一化行程
	Small Area High Gray Level Emphasis（SAHGLE，小区域高
	Gray Level Variance(GLV,灰度方差)

wavelet.HHH_glszm_GrayLevelVariance	wavelet.HLL_gldm_LargeDependenceEmphasis
0.77106195	-0.258908108
0.398131903	-0.038625604
0.580791511	-0.337771493
0.77106195	1.616341839
0.77106195	-0.309216859
-0.264854855	0.008115385
0.706317154	-1.501581434
0.655960074	-0.758705617
0.655960074	0.138404078
0.398131903	0.559098372
-0.264854855	1.963219052
0.677829438	-0.612654747
0.77106195	0.061989155
0.77106195	-0.325116147
0.77106195	1.394064273
0.77106195	-0.870157818
0.77106195	0.790793519
0.580791511	-0.663762124
0.398131903	0.331713077
0.77106195	-0.120319242
0.694010276	0.935503469
-1.559750843	0.435861919
0.188358751	1.65882825
0.580791511	0.502226541
0.77106195	0.017839859
0.398131903	3.607156773
-0.264854855	0.383299766
-0.264854855	0.028545464
0.443291419	0.794082053
-0.264854855	0.514353858
0.677829438	0.085236539
0.188358751	-0.579510961
-2.585308472	-0.154205954
0.398131903	-0.675641356
0.398131903	0.134494515
0.77106195	-3.104849182
0.77106195	-0.539924568
-0.264854855	-0.004788257
-0.264854855	-0.624470685
0.009980231	0.553613809
-0.264854855	-0.15750516
-0.264854855	-0.321896206
-0.264854855	0.102237941
-2.585308472	-0.067518807
-0.941371926	1.209375568
0.77106195	0.935152863
-0.264854855	-0.815145441
-0.720658238	0.35547933
-0.264854855	-0.494429962
0.188358751	0.027847206
0.77106195	-0.690265808

-1.559750843	-0.801779931
-0.264854855	-0.812467654
-1.259334955	1.07871712
0.398131903	0.59588125
-0.264854855	-0.081429623
-0.264854855	-0.454790448
0.77106195	0.376524459
0.77106195	0.22469487
0.77106195	-1.65411574
-0.264854855	-1.434809506
-0.264854855	-0.525305836
-0.264854855	1.023600521
-2.870833039	-0.659230325
0.398131903	1.096396679
-0.941371926	-1.456754907
-3.985698836	0.271146386
0.77106195	-0.423160923
0.188358751	-0.72950831
0.738801561	-0.25080872
0.77106195	1.254669245
0.77106195	-2.756942304
-2.585308472	1.001570765

呈不均匀性）
灰度强调）

elet.LHL_glrlm_RunLengthNonUniformityNorma	elet.LHL_glszm_SmallAreaHighGrayLevelEmph
1.175599648	-0.417526115
-0.214580978	-0.968330356
0.602374922	-0.762581175
-1.155936497	0.275429914
0.40484502	1.043130775
-0.503216688	-1.653006318
1.465941414	1.415210448
0.807218059	2.079689532
0.388677548	0.146209571
-1.125802779	0.097574507
-0.139825701	-0.186039314
0.827080955	0.076506601
-0.433892806	-2.067670216
0.168270451	2.722326236
-0.65370437	0.67203953
0.98933926	0.806345662
-0.498690116	-0.859405995
0.76728668	2.99249173
-0.950278876	-0.142388357
-0.165889824	2.042553279
-1.26237978	-0.083226559
-1.139870241	-0.117656731
-1.882920199	-1.166044228
-0.562430722	1.581247162
0.144639978	0.030218923
-3.132549768	-1.108714831
-0.190285193	0.955519712
-0.989353534	0.740483545
-0.991821777	1.484067942
-1.107916037	0.284378768
-0.239842311	1.126537229
-0.076633899	1.477852489
-0.191677467	0.188313358
0.244709404	-0.756541993
-1.264218342	0.339673232
2.990832492	-0.403943805
1.095221119	-0.692520821
-0.269811825	-0.066722048
0.956267443	-1.409956389
0.030496594	-0.743735507
-0.267984435	0.148485012
0.350454527	-0.780235778
-0.011444231	0.692266967
0.13111423	-0.218878031
-0.446890869	-0.301118272
-0.817782753	0.414216868
1.403939008	1.753611302
-0.030232495	-0.136081868
0.93757687	-1.024606928
0.044983546	-0.214963807
-0.583084977	0.037257014

1.195840797	-0.427310766
0.66346211	-1.263829789
-1.145951583	-0.78753523
0.44050311	-0.137126772
-0.194951731	-1.128488815
1.529286395	-0.32753378
0.161233472	0.325672054
-0.311611682	-0.702068764
1.09997206	-1.682974468
1.340661988	0.355186423
-0.576611945	-0.12415821
-1.477250849	-0.301802371
0.320165328	0.467770491
-0.991381279	-1.411709803
1.062766363	0.268043512
-0.340500032	-0.114053355
1.130074414	-0.751927161
-0.068393606	-0.142238767
0.847731164	0.057671641
-0.987284144	-0.263200161
2.42765756	-1.015771049
-0.751337585	-0.234356724

wavelet.LLH_gldm_GrayLevelVariance	Radscore_train
-0.460966626	-0.300220756
0.115042511	0.563182366
-0.608270566	-0.00898189
-0.769169317	-2.170151929
-0.576410057	-1.15646844
-0.006499673	2.118631743
1.479369773	0.435748238
0.79433337	-0.916734961
0.234347916	0.313070413
-0.881220265	-1.143014737
-0.55553529	-0.628760477
0.32556712	0.162225526
-0.857537837	0.263147167
-0.405899277	-2.067508426
-0.496943762	-1.61519201
-0.399853603	-0.700853151
0.372940892	0.244798231
-0.849935809	-2.110248381
0.017828725	-0.154538956
-0.013624729	-1.505786854
-0.905368614	-1.95197456
-0.364512595	2.142874557
-1.27516107	-0.965819441
-0.449382367	-1.758717247
0.050647124	0.158821561
-1.473095352	-3.065412721
-0.7722891	0.24627471
-0.785388826	-0.40298703
-0.58516979	-1.465983327
0.793152597	0.516657454
0.600707715	-0.795580917
0.06108955	-0.49079358
-0.610869779	3.598054007
1.819524024	1.994749932
-0.361709566	-0.402522917
2.773471056	5.502092536
1.014164453	1.19952952
-0.375667885	1.126564243
1.525766078	2.835420822
0.595470954	1.250041422
-0.564761265	0.789793839
-0.222277022	1.241753938
-0.457621207	0.382345788
-0.248461543	4.292804928
-1.259547221	0.433002022
0.4328168	-1.135870317
0.935563492	1.286499516
-0.222447927	1.572445
-0.763780072	1.581433882
1.363945396	1.141767416
-0.73985607	-0.736433767

0.049812821	3.652904646
1.154391151	4.42886853
-0.963771821	1.474376222
-0.408066917	0.138954687
-0.640686261	1.524490977
-0.261902045	1.404562685
0.507017384	-0.670057683
0.650747946	-0.019010195
1.578048203	3.858967246
2.008348398	2.861131759
0.739369306	1.336690787
0.122804151	0.931860539
0.94374313	4.795093797
-1.58453351	0.297499101
0.335373028	2.875881779
0.961552669	6.344019772
-1.087030428	0.720703974
-0.691821329	-0.171048374
3.840139833	2.117690761
-1.067712888	-2.041629254
-0.954359741	2.267313378
-1.217978543	3.025164598
